# Gastroduodenal intussusception due to gastrointestinal stromal tumor

**DOI:** 10.1002/ccr3.1786

**Published:** 2018-09-12

**Authors:** Utpal De, Srijan Basu

**Affiliations:** ^1^ Department of Surgery Bankura Sammilani Medical College Bankura West Bengal India

**Keywords:** GIST intussusception

## Abstract

Gastric GIST should be kept in mind in patients with gastric outlet obstruction.

## 1 QUIZ

**Figure 1:**
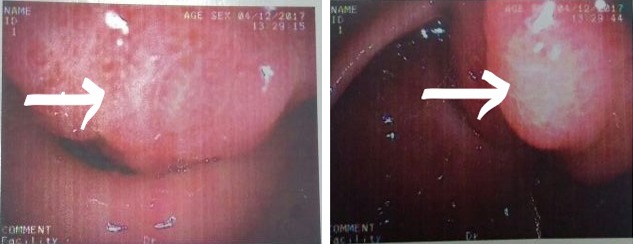
Endoscopy showing submucosal tumor in the antrum with mucosal ulceration (white arrowhead)

**Figure 2:**
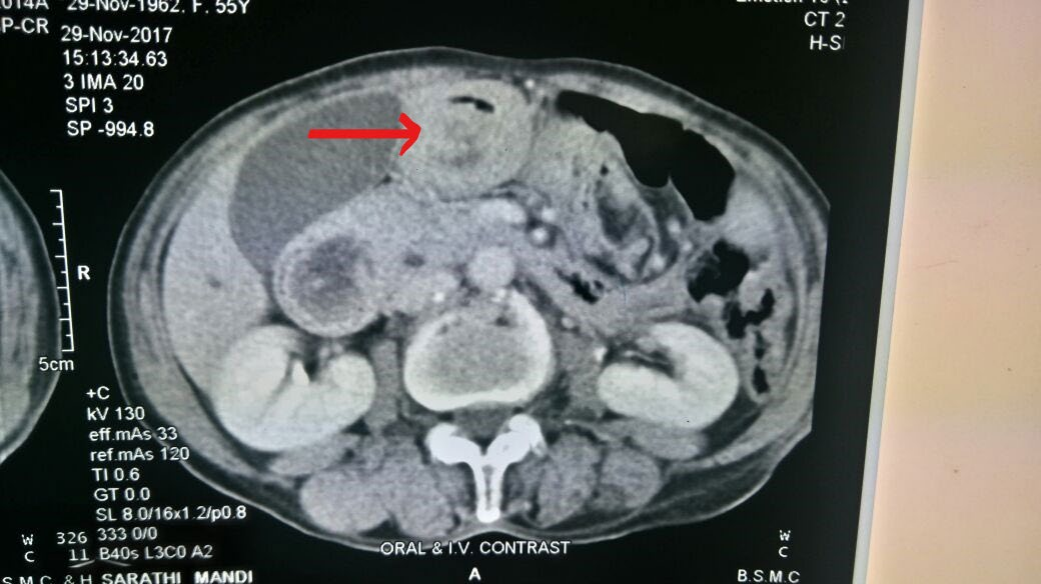
CT scan showing gastric tumor invaginating into second part of duodenum (red arrowhead)

**Figure 3:**
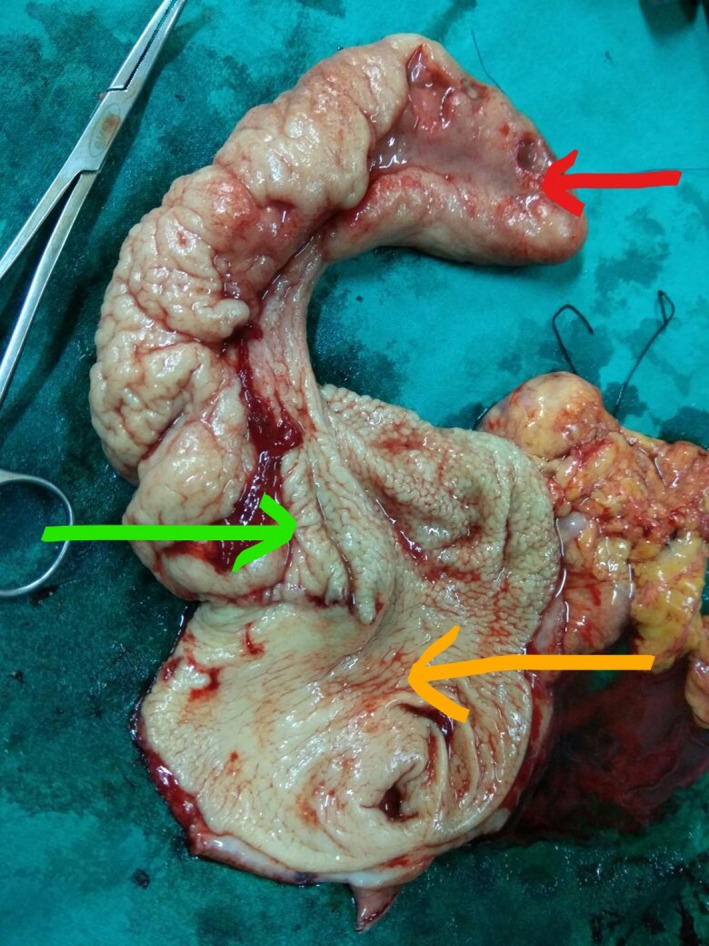
Resected specimen of GIST showing apex (red arrow), base (green arrow), and part of cuff of stomach (yellow arrow)

A 42‐year‐old female presented with symptoms of upper abdominal pain and intermittent vomiting after meals for the past 6 months. She did not have any significant past history. She presented with clinical features of acute gastric outlet obstruction. Blood investigations showed anemia. Endoscopy (Figure 1) revealed a submucosal tumor from the anterior wall of stomach with central ulceration prolapsing into the duodenum. CT scan (Figure 2) demonstrated 8 × 7 × 4 cm sized heterogeneously enhancing pedunculated polypoid mass attached to the antropyloric region, lying within the duodenum extending till its third part. Laparotomy was performed, and the mass was removed with a cuff of anterior wall of stomach with GIA stapler. Postoperative period was uneventful, what is the lesion in the resected specimen of stomach? (Figure 3).

## 2 ANSWER

Gastroduodenal intussusception (10%)^1^, ^2^ causing acute gastric outlet obstruction due to pedunculated gastric gastrointestinal stromal tumor (GIST) is rare. GIST (mesenchymal tumor) is pathologically defined by positive immunostaining for c‐kit proto‐oncogene—CD117 (95%) and CD34 (60%‐70%).^2^ Ulceration of the apical mucosa results in bleeding (50%).^2^ Endoscopy and CT scan are diagnostic. Treatment for localized GIST is complete surgical resection. Fletcher's risk stratification indices include tumor size, mitotic index, nonradical resection (R1), and tumor rupture.^1^, ^2^ Postoperative chemotherapy improves relapse‐free survival, but overall survival remains unchanged.

## CONFLICT OF INTEREST

None declared.

## AUTHOR CONTRIBUTION

UD and SB: conceived of the presented idea. UD: encouraged SB to investigate and supervised the findings of this work. All authors: discussed the results and contributed to the final manuscript.
